# Supplementary figures and images for: Waste Coffee Ground Biochar: A Material for Humidity Sensors
Source: Sensors (Basel). 2019 Feb 15;19(4):801. doi: 10.3390/s19040801 (PMC6412531; doi:10.3390/s19040801)

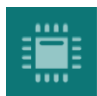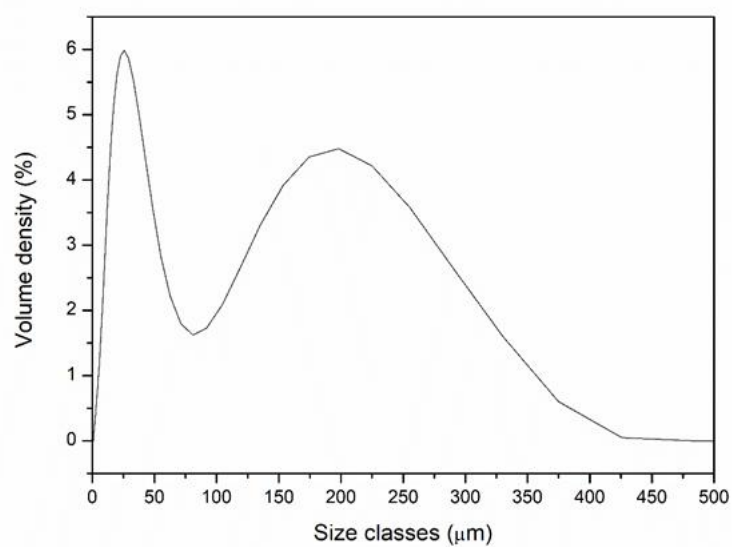

**Figure S1.** Particle size distribution of CGB powder after 5 min of sonication in ethanol.

Supplement: Supplementary file 1 [file sensors-19-00801-s001.pdf]
